# Are more exercise components in combined cognitive and physical training better for older adults?: A systematic review and network meta-analysis of randomized controlled trials

**DOI:** 10.1097/MD.0000000000041572

**Published:** 2025-02-21

**Authors:** Conglin Han, Dan Zhang, Weishuang Sun, Jiawei Liu, Ruifeng Sun, Weijun Gong

**Affiliations:** a Beijing Rehabilitation Hospital, Capital Medical University, Beijing, China; b Rehabilitation Medicine Academy, Shandong Second Medical University, Shandong, China; c Beijing Rehabilitation Medicine Academy, Capital Medical University, Beijing, China; d Department of Neurological Rehabilitation, Beijing Rehabilitation Hospital, Capital Medical University, Beijing, China.

**Keywords:** cognitive and physical training, executive function, memory, older adults

## Abstract

**Background:**

Age-related cognitive problems are becoming increasingly prevalent in older adults; thus, maintaining normal cognitive abilities and delaying cognitive decline are essential for promoting healthy aging.

**Methods:**

This systematic review and network meta-analysis examined the effects of combining cognitive and physical training by including various exercise components to improve memory and executive function in older adults. The Cochrane Risk Assessment Tool was used to assess the risk of bias in the included literature. Pairwise meta-analysis was conducted using RevMan 5.3, while network meta-analysis was performed with Stata 15.1, and interventions were ranked based on the results.

**Results:**

A total of 8180 articles were screened, and 16 randomized controlled trials were included. Pairwise meta-analysis showed that cognitive-physical interventions with 2 (SMD = 0.26, 95% CI: 0.07–0.44, *P* = .007) and 3 or more exercise components (SMD = 0.43, 95% CI: 0.13–0.73, *P* = .004) significantly improved memory compared to controls. For executive function, both interventions with 2 (SMD = 0.40, 95% CI: 0.15–0.65, *P* = .002) and 3 or more exercise components (SMD = 0.55, 95% CI: 0.16–0.93, *P* = .005) outperformed the control group. Network meta-analysis confirmed that interventions with 2 (SMD = 0.24, 95% CI: 0.06–0.42) and 3 or more components (SMD = 0.43, 95% CI: 0.13–0.73) improved memory, while a single exercise component was most effective for executive function (SMD = 1.27, 95% CI: 0.05–2.49). Overall, we demonstrated that combined cognitive-physical intervention training with multiple exercise components significantly improved memory and executive function compared to controls. The effects of combined training with 3 or more exercise components were likely the most effective in improving memory.

**Conclusion:**

Cognitive-physical combined interventions are increasingly applied in clinical research on age-related cognitive decline. Our meta-analysis indicates that interventions incorporating multiple exercise components are more effective than those with a single exercise component. These findings provide a basis for future cognitive and physical interventions for older adults and can inform the design of effective intervention programs.

## 1. Introduction

The aging population continues to rise globally at an accelerated rate with a burgeoning life expectancy.^[1]^ Nonetheless, age-related impairments and diseases are becoming increasingly prevalent. On average, 16% to 20% of older adults are afflicted with either disease or dysfunction, possibly leading to a gradual loss of normal physiological function.^[2]^ According to the World Health Organization, cognitive impairment and dementia have emerged as the primary causes of disability among the older population.^[3]^

Currently, evidence is lacking regarding the efficacy of drug therapies for cognitive deficits associated with dementia.^[4]^ Consequently, nonpharmacological interventions have emerged as an area of interest among researchers. Among these, cognitive training has a notable enhancing effect on cognitive function (particularly memory, executive function, and global cognitive function) in older adults with and without cognitive impairment.^[5,6]^ Similarly, physical exercises for improving cognitive function in older individuals have been proven beneficial.^[7,8]^ These nonpharmacological interventions are endorsed by relevant clinical recommendations and practice guidelines.^[4,9,10]^

Based on the benefits of cognitive training and physical exercise, an increasing number of studies have developed and used a new type of treatment strategy that combines cognitive training and physical exercise – cognitive-physical intervention (also known as cognitive-physical or dual-task training by some researchers). Several meta-analyses have confirmed that the combined non-pharmacological intervention of cognitive training and physical exercise produces better outcomes than either cognitive training or physical exercise alone.^[11,12]^ It has been suggested that combining cognitive and physical training may synergistically improve cognitive function and brain structure.^[13]^

In recent years, we have observed an increasing complexity in the form of cognitive-physical combined interventions. This complexity is primarily manifested in the incorporation of multiple types of physical exercise, rather than a single modality, in conjunction with cognitive training. For example, in the study by Park et al, the cognitive-physical combined intervention included 2 exercise components: aerobic and strength training.^[14]^ Previous meta-analyses have shown that multi-component exercise has a positive effect on the cognitive function of elderly individuals with mild cognitive impairment (MCI).^[15]^ Network meta-analyses have further confirmed that multi-component exercise may be the most effective type of intervention for improving both global cognitive function and executive function in middle-aged and older adults with cognitive impairments.^[16]^ However, no studies have yet specifically addressed the impact of cognitive-physical combined interventions with multiple exercise components on cognitive function in the elderly. Therefore, we conducted this network meta-analysis to explore the effects of cognitive-physical combined interventions, incorporating varying numbers of exercise components, on cognitive function in older adults with or without mild cognitive dysfunction.

## 2. Method

The Preferred Reporting Items for Systematic Reviews and Network Meta-Analyses (PRISMA-NMA) was used to guide this systematic review and network meta-analysis. This study protocol is registered with PROSPERO (CRD42023452861).

### 2.1. Eligibility criteria for study selection

*Population:* Adults aged ≥ 60 years with cognitive health or mild cognitive dysfunction, including subjective cognitive decline and mild cognitive impairment.

*Intervention:* Any intervention combining cognitive training with physical exercise in the abovementioned population. Cognitive training was defined as the repetitive practice of tasks targeting one or more cognitive domains. Physical training included any form of organized physical activity, such as aerobic, strength, or functional (i.e., gait or balance) training. Dual-task training was either performed simultaneously or sequentially.

*Comparison:* A control group mainly included participants involved in health education, socialization, and activities of daily living who did not engage in cognitive or physical interventions.

*Outcomes:* The studies’ results reported at least 1 effect of the interventions on memory and executive function.

*Study:* Only randomized controlled studies were included in this review. Studies including participants with psychiatric disorders, traumatic brain injury, and neurological disorders were excluded, as these conditions are known to significantly affect cognitive function and may introduce confounding variables that could lead to biased results. Studies with missing information, duplicate publications, and written in a language other than English were also excluded.

### 2.2. Search strategy

We systematically searched 3 electronic databases, including PubMed, Cochrane Library, and Embase, from inception to September 2023. A search strategy combining subject terms and free words was used to obtain better search results. Specific search strategies for PubMed, Cochrane Library, and Embase are described in File S1, Supplemental Digital Content, http://links.lww.com/MD/O406. Excluding the control group, we categorized the cognitive-physical interventions into 3 groups: cognitive-physical interventions with only 1 exercise component (hereinafter referred to as combined intervention 1); cognitive-physical interventions with 2 exercise components (hereinafter referred to as combined intervention 2); and cognitive-physical interventions with 3 or more exercise components (hereinafter referred to as combined intervention 3).

### 2.3. Study selection

Two reviewers (C.L.H. and D.Z.) independently screened records by titles and abstracts against the pre-determined eligibility criteria. After removing duplicates, the remaining records were re-reviewed to validate the eligibility for inclusion. The original authors were contacted in the event of missing or unavailable data. Any screening controversies were resolved after consensus with a third reviewer (W.J.G.).

### 2.4. Risk of bias

We used the Cochrane Risk of Bias Assessment Tool, as detailed in the Cochrane Handbook (https://training.cochrane.org/handbook/archive/v5.1/), for impartial evaluation of the risk of bias involved in the included randomized controlled trials. The parameters listed in the Cochrane Handbook incorporated randomized sequence generation, allocation concealment, blinding, incomplete data, and selective reporting, with each classification being divided into categories of low, unclear, and high risks. Each trial was evaluated independently by 2 reviewers. Any discrepancy in agreement was resolved after consensus with a third reviewer.

### 2.5. Quality of evidence assessment

We assessed the certainty of the combined evidence for each outcome according to the framework described by Salanti et al. (based on the GRADE framework).^[17]^ We employed the CINeMA (Confidence in Network Meta-Analysis) web application for implementation.^[18]^ The application categorizes confidence levels as high, medium, low, and very low. We checked the credibility of all comparisons.

### 2.6. Data extraction

Two researchers (C.L.H. and D.Z.) independently extracted data from each included study using a standardized form, including details on study characteristics such as the first author, year of publication, cognitive state, sample size, age, gender (female), physical intervention, combination mode, duration per session, frequency, total intervention time, information on the control group, intervention intensity, participant adherence, and results. They also made efforts to contact the original authors via email to obtain any missing or essential information. Any disagreements between the researchers were resolved through discussion until a consensus was reached.

### 2.7. Statistical analysis

All pairwise meta-analyses were conducted via the RevMan 5.3 software. The alterations in cognitive function (memory and executive function) were assessed using means and standard deviations. If these measurements were unreported, we derived them via the initial mean and standard deviation. The Cochrane Handbook for the Systematic Evaluation of Interventions stipulates the formula for calculating such values (https://training.cochrane.org/handbook). The random-effects model was used in the pairwise meta-analysis because it better handles potential between-study variability, especially when the number of studies is small, and provides more robust effect estimates. To account for variations in assessment tools, standardized mean difference (SMD) and 95% confidence intervals were used to report combined effect sizes. As all of the outcomes of interest were continuous, but could be measured on different scales, SMD was used as the effect estimate. Heterogeneity was measured using the *I*^2^ statistic. As per the Cochrane Handbook, heterogeneity was classified into 4 categories: insignificant (0–40%), moderate heterogeneity (30–60%), significant heterogeneity (50–90%), and extremely significant heterogeneity (75–100%). A random-effects model was used to indirectly control for confounding factors. Sensitivity analysis was conducted when *I*^2^ was >50%.

We used Stata 15.1 software to conduct a frequency-based random effects network meta-analysis of direct and indirect evidence. Comparative relationships between different interventions were demonstrated by using network plots. When a closed loop of evidence in a network graph was observed, we conducted the inconsistency analysis and a statistical assessment of global consistency, i.e., agreement between direct and indirect evidence, using the “loop-specific” approach. Then, direct evidence was separated from indirect evidence by node splitting. Global inconsistency was determined at a *P* value > .05.

The inconsistency factor was close to zero, indicating a lack of local inconsistency. SMDs and their corresponding 95% confidence intervals were utilized to evaluate the relative effectiveness of the interventions. Interventions are represented by dots in the network plot, with larger dots indicating more patients receiving the intervention. A straight line signifies a direct comparison between 2 interventions, while the thickness of the line segment reflects the number of studies with such direct comparisons. The study utilized the surface under the cumulative ranking curve (SUCRA) to indicate the probability ranking (0% ≤ SUCRA ≤ 100%). A higher SUCRA value suggests a more effective intervention. Comparison-adjusted funnel plots were implemented to investigate small sample effects and publication bias. The symmetric distribution of data points around the central line indicated no bias.

## 3. Results

### 3.1. Search results

**Figure 1.**
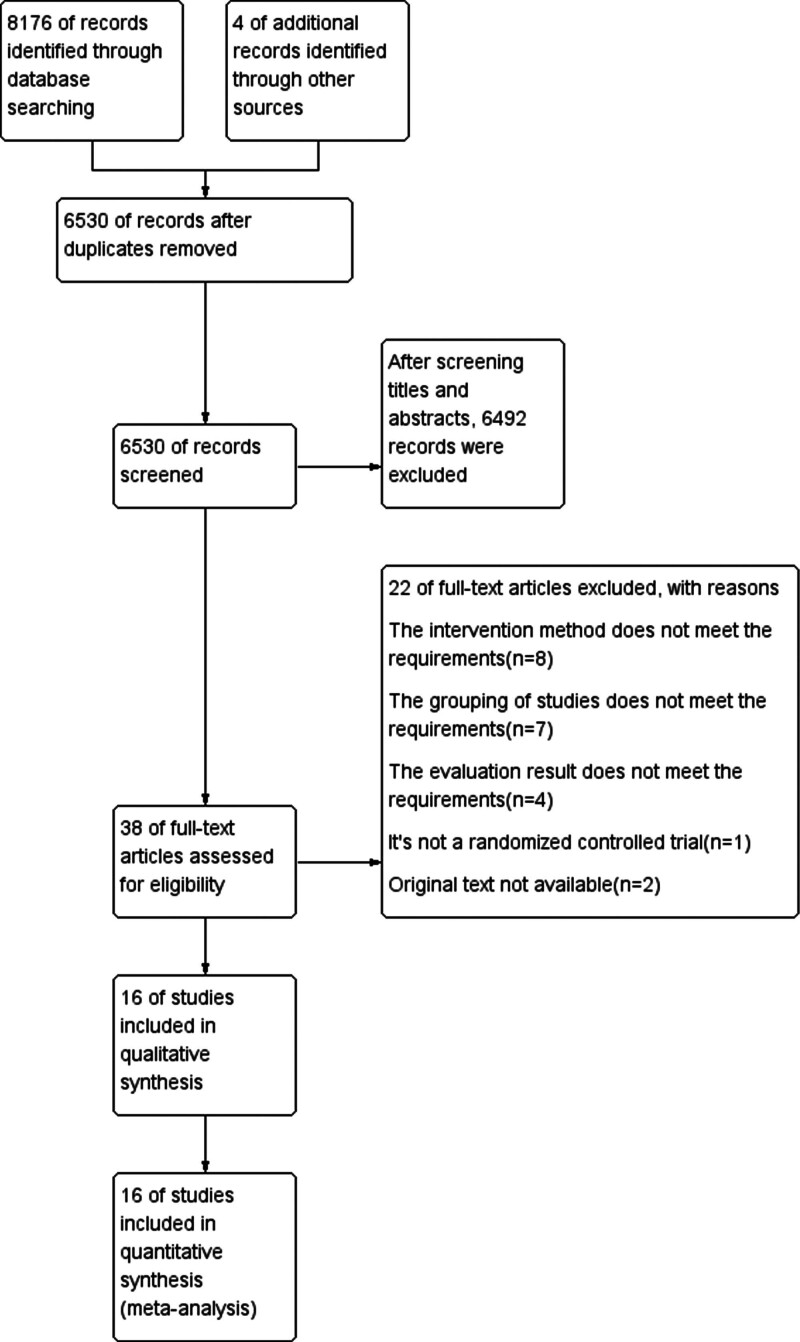
Study flow diagram.

Figure 1 illustrates the process of study selection. During title and abstract screening, 6492 articles were excluded. At the outset, 8180 articles were included, of which 6530 were retained following the removal of duplicates. Screening of full-text articles led to the inclusion of 16 studies from the remaining 38.

### 3.2. Characteristics of included randomized controlled trials

A total of 16 randomized controlled trials were included in this review, published between 2002 and 2023. The characteristics of all included studies are summarized in Table 1. The studies were conducted in different countries: France (n = 2), USA (n = 3), Japan (n = 2), Germany (n = 1), Australia (n = 1), Hong Kong, China (n = 1), Canada (n = 1), Iran (n = 2), South Korea (n = 1), and Switzerland (n = 1). A single multicenter study was conducted in different countries, including Italy, Greece, Spain, and Serbia. A total of 1149 participants were included in 16 studies, with sample sizes ranging from 16 to 263. Of all the included studies, the use of combined intervention with 1, 2, and 3 exercise components was reported by 5,^[19–23]^ 6,^[14,24–28]^ and 4 studies,^[29–32]^ respectively. The remaining article compared the effects of combined interventions between 1 and 2 exercise components.^[33]^ The total duration of all interventions ranged from 8 to 52 weeks, with a frequency of 30 to 110 minutes per session and 1 to 6 times per week. The intervention intensity (exercise intensity) varied from low to high, with 5 studies failing to report specific details. Regarding participant adherence (defined as the degree to which participants complied with and completed the prescribed intervention plan), 3 studies did not provide clear information, 1 study reported adherence < 80%, and the remaining studies reported adherence rates > 80%.

**Table 1:** Characteristics of selected randomized control trials

Study	Nation	Participants				Physical intervention	Combination mode	Duration per session	Frequency (times/week)	Total intervention time (week/hour)	Control group	Intervention intensity (exercise intensity)	Participant adherence	Result	
		Cognitive state	Sample size	Age	Gender (female %)									Memory function	Executive function
Combined intervention 1															
Combourieu Donnezan, Laure 2018^[19]^	France	MCI (MMSE TG:28.1 ± 0.36 CG:27.3 ± 0.5)	TG: 21 CG:14	TG: 75.2 ± 1.3 CG:79.2 ± 4	–	Aerobic exercise	Simultaneous	1	2	12/24	CG: Maintain a routine	Moderate intensity (60% of maximal heart rate)	84%		Stroop color word test; digit span; matrix reasoning test
C. Fabre 2002^[20]^	France	Cognitively Healthy	TG: 8 CG:8	TG: 64.9 ± 1.4 CG: 65.7 ± 1.5	TG: 12.5% CG: 12.5%	Aerobic exercise	Sequence	CT: 90 PE: 60	3 (CT: 1, PE: 2)	8/28	CG: Leisure activities	Low intensity to high intensity	– (very good)	Clinical scale of Wechsler (Logical memory-immediate recall)	clinical scale of Wechsler (Digit span)
Lam, L C 2015^[21]^	Hong Kong, China	MCI (MMSE TG:25.2 ± 2.2 CG:25.6 ± 2.4)	TG: 132 CG:131	TG:76.3 ± 6.6 CG: 75.4 ± 6.1	TG: 78.79% CG:77.86%	Mind body exercise (e.g., Tai Chi)	Sequence	60	3	52/156	CG: Social Activities	-	65%	List learning delayed recall test for Episodic memory	Category Verbal Fluency Test.
McDaniel, M A 2014^[22]^	USA	Cognitively health (MMSE TG:29 ± 1 CG:29 ± 1)	TG: 24 CG:25	TG: 65 ± 6 CG: 64 ± 7	TG: 13% CG: 15%	Aerobic exercise	Sequence	60 (PE)	6 (CT: 3, PE: 3)	24(PE: 24, CT: 8)/96	CG Low-intensity exercise, health education	Low intensity to high intensity (The heart rate reserve gradually increases from 50-60% to 65-85%)	96%	Memory for Health Information	
Norouzi, E 2019^[23]^	Iran	Cognitively health (MMSE TG:26.67 ± 2.56 CG:25.83 ± 2.43)	TG:20 CG:20	TG: 68.51 (3.65) CG: 68.10 (3.71)	–	Resistance training	Simultaneous	60-80	3	4/14	CG: conference	–	–		N-back
Combined Intervention 2															
Barban, F 2017^[24]^	Italy, Greece, Spain and Serbia	Cognitively healthy	TG: 121 CG: 123	TG: 74.5(7.9) CG: 76(8.8)	TG:72% CG: 65%	Balance and gait training	sequence	60 (CT: 30, PE: 30)	2	12/24	CG: Computer data entry	–	–	RAVLT, ROCF	
Callisaya, M L 2021^[25]^	Australia	Subjective cognitive decline or MCI (MMSE TG:24.6 ± 2.6 CG:24.4 ± 3.1)	TG:44 CG: 49	TG: 72.9 7.2 CG: 72.8 6.9	TG:61% CG: 55%	Balance and strength training	Simultaneous	–	–	26/52	CG: Get health information	–	85%	Hopkins Verbal Learning test.	Digital Symbol Coding test; Stroop test; TMT
Legault, C 2011^[26]^	USA	Cognitively Healthy(at risk of cognitive decline) (3MSE TG:94.6 ± 4.3 CG:94.3 ± 2.3)	TG: 19 CG: 18	TG: 76.9 (4.0) CG: 75.4 (4.8)	TG: 63% CG: 38%	Aerobic and flexibility exercise	Sequence	105 (CT: 45, PE: 60)	2	16/56	CG: Health education	–	90%	Hopkins Verbal Learning test; Wechsler Memory Scale-III	N-back; TMT; Task Switching; Self-Ordered Pointing Task; Flanker Task
Linde, K 2014^[27]^	Germany	Cognitively healthy	TG: 16 CG: 13	TG: 65.59 (3.74) CG: 66.56 (3.20)	TG: 65% CG: 63%	Aerobic and Strength exercise	Sequence	90 (CT 30, PE: 60)	1	16/24	CG: Maintain a routine	Low to moderate intensity (The heart rate reserve gradually increases from 40-50% to 60-70% %)	81%	Word List test	Digit-symbol substitution test, TMT
Nishiguchi, S 2015^[28]^	Japan	Cognitively healthy (MMSE TG:27.4 ± 1.8 CG:27.9 ± 2.0)	TG: 24 CG: 24	TG: 22 CG: 25	TG: 73.0 ± 4.8 CG: 73.5 ± 5.6	Strength and stepping exercise	Simultaneous	90	1	12/18	CG; Without any intervention	Moderate intensity	92%	WMS-R	TMT
Park, H 2019^[14]^	Korea	MCI (MMSE TG:24.6 ± 2.6 CG:24.4 ± 3.1)	TG:25 CG: 24	TG: 70.55 ± 6.46 CG: 72.76 ± 5.37	TG:60% CG: 24	Aerobic and balance training	Simultaneous	110	1	24/44	CG: Maintain a routine	Moderate to high intensity (55% to 80% of maximum heart rate)	95%		Symbol–Digit Substitution Test; TMT; Digit Span
Combined intervention 3															
Adcock, Manuela 2020^[29]^	Switzerland	Cognitively healthy (MMSE TG:28.9 ± 1.1 CG:29.2 ± 0.9)	TG: 15 CG: 16	PE: 77.0 ± 6.4 CG: 70.9 ± 5.0	PE: 66.7% CG: 37.5%	Strength, balance, movement training	Simultaneous	30–40	3	16/28	CG: Maintain a routine	Moderate intensity (approximately 60% of estimated maximum heart rate)	100%	WMS-R	Stroop test; TMT
Damirchi, A NC 2018^[30]^	Iran	MCI (MMSE TG:23.30 ± 1.84 CG:23.44 ± 2.06)	TG: 13 CG: 9	TG: 67.76 (4.69) CG: 69.11 (4.93)	TG: 100% CG: 100%	Aerobic exercise, muscular strength and movements	Sequence	60 (CT: 30, PE: 30) – 120 (CT: 60, PE: 60)	3	8/36	CG: Maintain a routine	Moderate to high intensity (gradually increasing from 55% to 75% of heart rate reserve)	81%		Stroop test; Digit span
Shatil, E 2013^[31]^	USA	Cognitively healthy	TG: 29 CG: 29	TG: 79 ± 5.49 CG:81 ± 5.25	TG: 69% CG: 65.5%	Aerobic, Strength and flexibility exercise	Sequence	CT: 40 PE: 45	6 (CT: 3, PE: 3)	16/68	CG: Book club	Low to moderate intensity	100%	CogniFit neuropsychological evaluation (global visual memory)	CogniFit neuropsychological evaluation (speed of visual-spatial information processing)
Suzuki, T 2013^[32]^	Japan	MCI (MMSE TG:26.8 ± 2.3 CG:26.3 ± 2.7)	TG: 47 CG: 45	TG: 74.8 ± 7.4 CG: 75.8 ± 6.1	TG: 50% CG: 48%	Aerobic, Strength balance, dual task training)	Simultaneous	90	2	26/78	CG: Health education	Moderate intensity (approximately 60% of maximum heart rate)	86%	WMS-R	
Combined intervention 1 and Combined intervention 2															
Desjardins-Crépeau, L 2016^[33]^	Canada	Cognitively healthy (MMSE TG:28.8 ± 1.3 CG:29.4 ± 0.7)	TG1: 22 TG2: 20	TG1: 72.7 (7.4) TG2: 73.2 (6.3)	TG1: 59% TG2: 85%	TG1: Aerobic and resistance exercise TG2: Stretch Exercise	Sequence	60	3 (PE: 2,CT: 1)	12/36	–	Moderate to high intensity	100%	RAVLT	Stroop test; TMT

3MSE = Modified Mini Mental State Exam, CG = control group, Combined Intervention 1 = cognitive-physical interventions with only one exercise component, Combined Intervention 2 = cognitive-physical interventions with 2 exercise components, Combined Intervention 3 = cognitive-physical interventions with 3 or more exercise components, MMSE = Mini-Mental State Examination, RAVLT = Rey Auditory Verbal Learning test, ROCF = Rey-Osterrieth Complex Figure, TG = Treatment Group, TMT = Trail Making Test, WMS-R = Wechsler Memory Scale Revised.

### 3.3. Risk assessment of bias in literature

**Figure 2.**
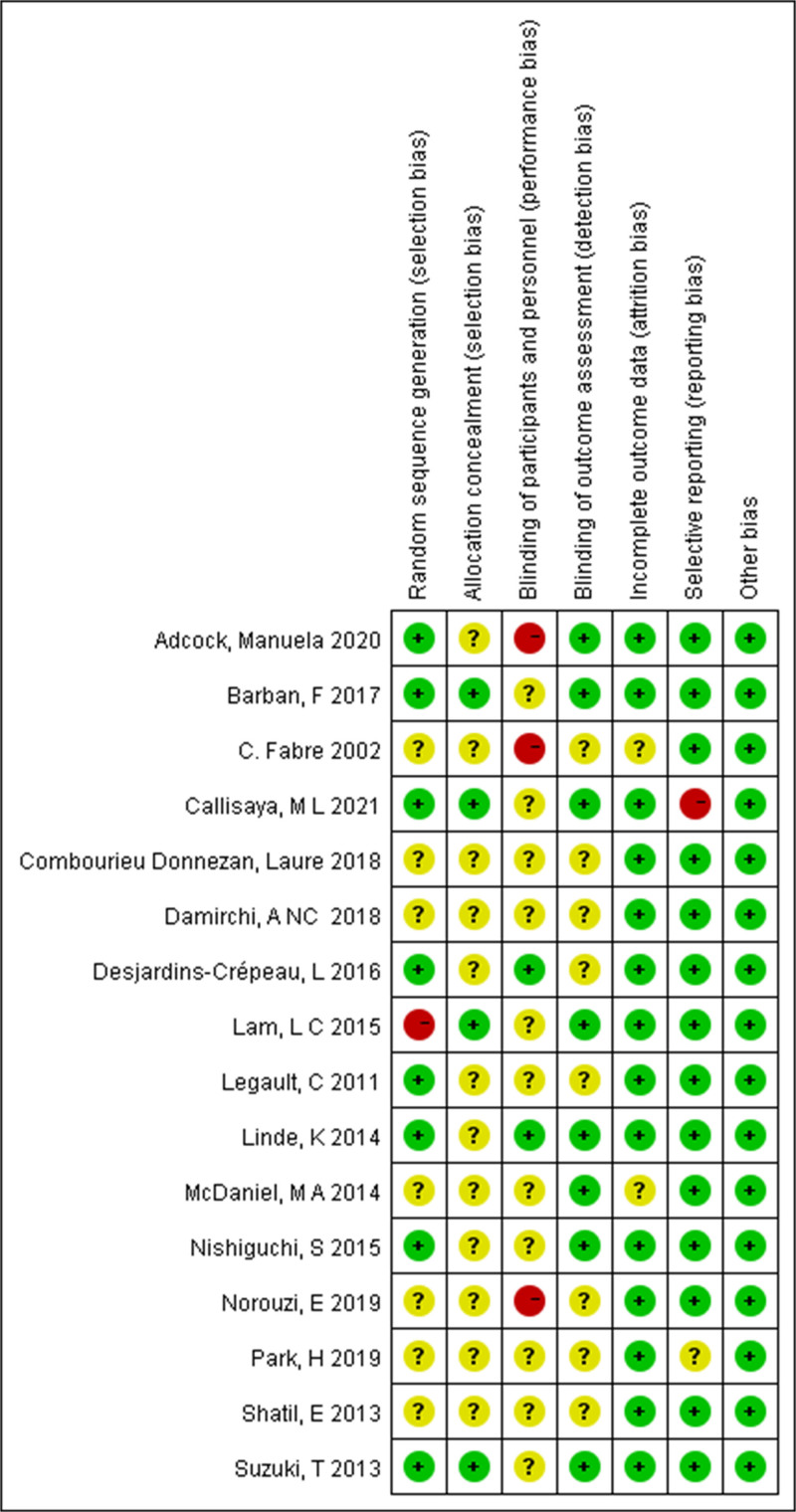
Risk of bias assessment in the included studies.

In 50% (8/16) of these studies, the method of random sequence generation needed to be described in detail or had apparent flaws. The risk of bias for these included studies is shown in Figure 2. Seventy-five percent (12/16) of the studies did not clearly describe the allocation concealment method. Participants and researchers were blinded in only 12.5% (2/16) of the studies. Fifty-six percent (9/16) of the studies were blinded to the outcomes assessment. Lost visit bias and reporting bias were low risks in 87.5% (14/16) of these studies.

### 3.4. Pairwise meta-analysis results

**Figure 3.**
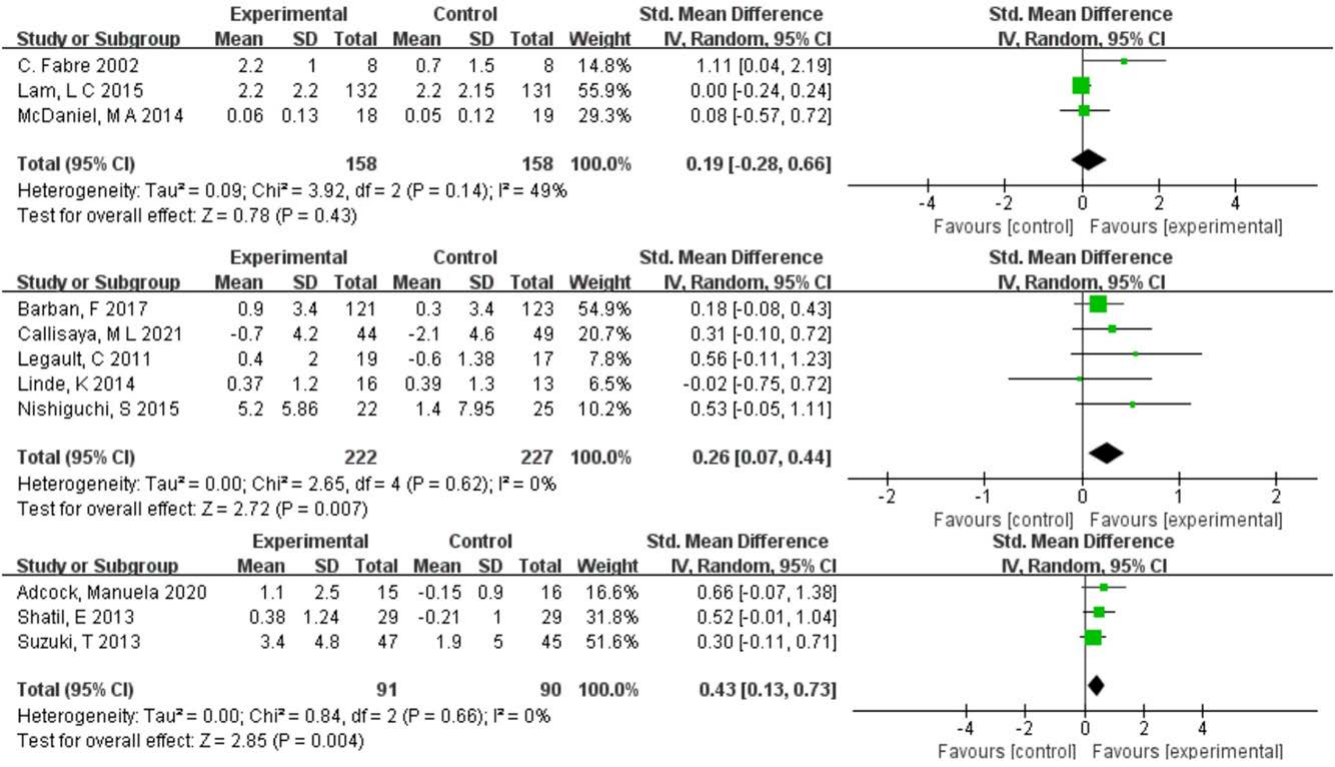
Forest plot of the results of the meta-analysis showing relative effects of the 3 interventions on memory function (top: combined intervention 1, middle: combined intervention 2, bottom: combined intervention 3).

**Figure 4.**
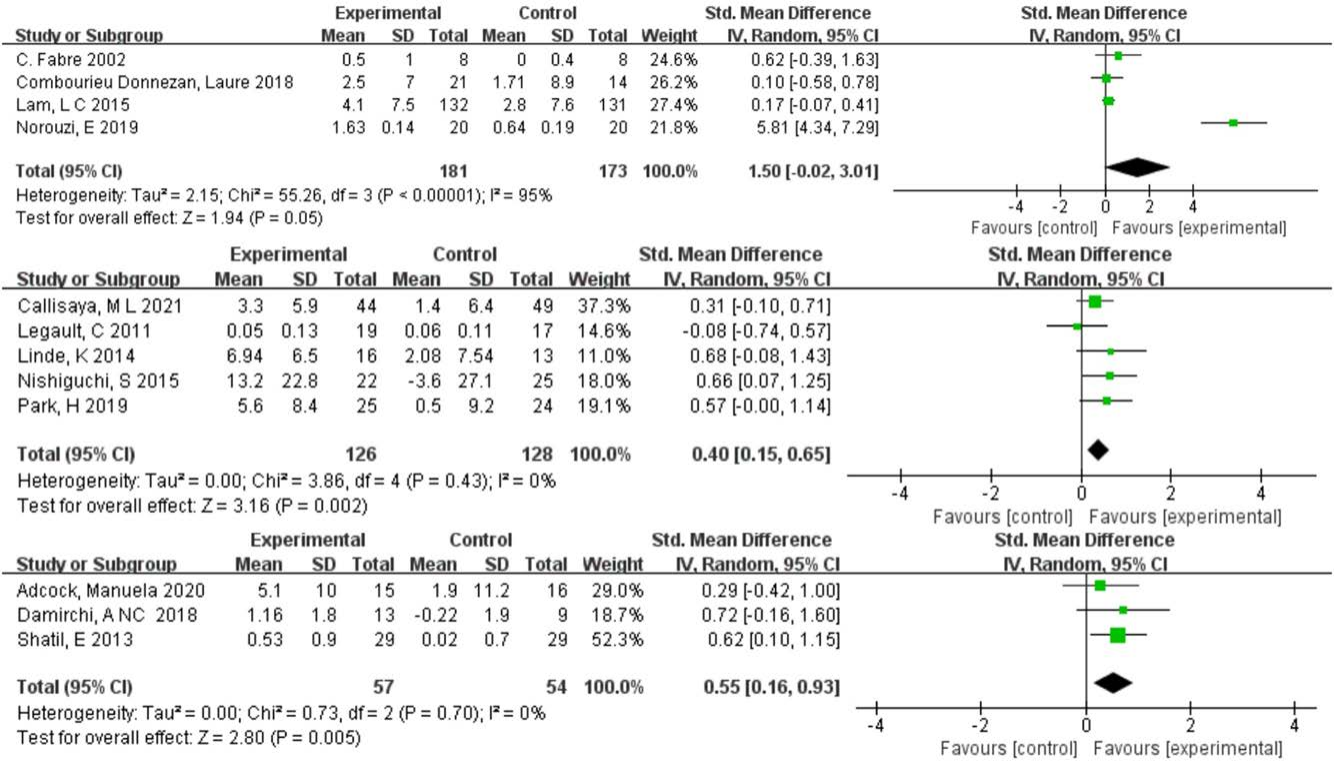
Forest plot of the results of the meta-analysis showing relative effects of the 3 interventions on executive function. (top: combined intervention 1, middle: combined intervention 2, bottom: combined intervention 3).

In a pairwise meta-analysis, we used a random effects model to assess directly comparable effects of a combined intervention with different exercise components on memory and executive function. The findings are displayed as forest plots in Figures 3 and 4. The meta-analysis results for memory function indicate that combined intervention 2 (SMD = 0.26, 95% CI: 0.07–0.44, *P* = .007) and combined intervention 3 (SMD = 0.43, 95% CI: 0.13–0.73, *P* = .004) differed significantly from the control group. In the meta-analysis of executive function outcomes, both combined intervention 2 (SMD = 0.40, 95% CI: 0.15–0.65, *P* = .002) and combined intervention 3 (SMD = 0.55, 95% CI: 0.16–0.93, *P* = .005) were significantly more effective in improving executive function than the control group.

### 3.5. Sensitivity analyses

Across all outcomes, we found a high degree of heterogeneity in the effectiveness of combined intervention 1 on executive function (*I*^2^ = 95%, *P* for *I*^2^ < .00001). Sensitivity analyses were performed on the above results (File S2, Supplemental Digital Content, http://links.lww.com/MD/O407). Regarding the effectiveness of combined intervention 1 on executive function, heterogeneity was significantly reduced after omitting the study by Norouzi et al, which was conducted in 2019^[23]^ (*I*^2^ = 0%, *P* for *I*^2^ = 0.67).

### 3.6. Results of the network meta-analysis

**Figure 5.**
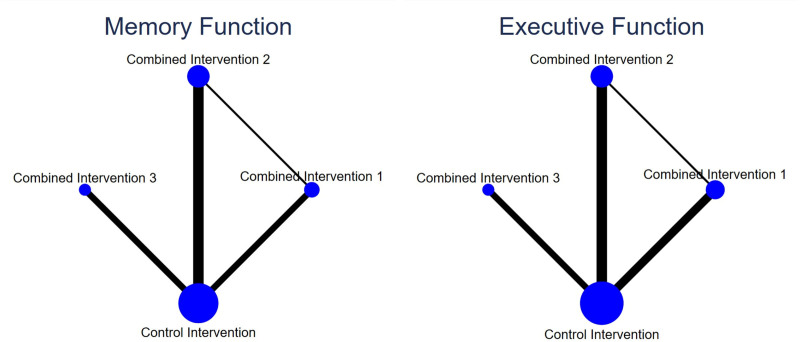
(left) network map-memory function; (right) network map-executive function.

We performed a network meta-analysis of memory and executive function, and the corresponding network maps are shown in Figure 5. The network maps for the 2 outcomes were similar, with the control intervention having the highest sample size, while the remaining 3 combined interventions have relatively smaller sample sizes. Direct comparisons were mostly made between combined interventions with different exercise components and controls, with very few direct comparisons between various exercise components. Only the control intervention, combined intervention 1, and combined intervention 2 formed a closed loop. To assess consistency between direct and indirect comparisons, we employed both global and local consistency analyses. No statistically significant differences between the results of global consistency analyses for memory function (Chi-square = 0.34, *P* = .5602) and for executive function (Chi-square = 0.35, *P* = .5514). Neither the results of the local consistency analyses of memory function nor the executive function (inconsistency factor close to 0) were statistically different (File S2, Supplemental Digital Content, http://links.lww.com/MD/O407).

**Figure 6.**
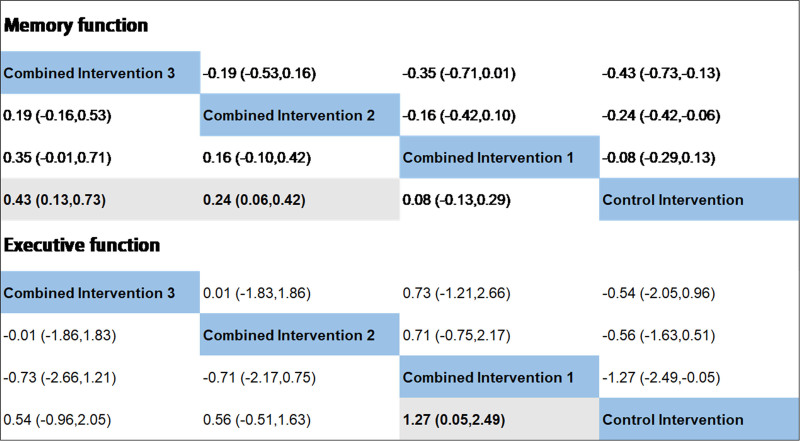
Comparative effects of various interventions.

For the network meta-analysis of memory function, 12 out of 16 two-arm studies were included. combined intervention 1, combined intervention 2, and combined intervention 3 were compared with a control group in 5, 3, and one study, respectively. The comparative effectiveness of the various interventions in the network meta-analysis showed (Fig. 6) that combined intervention 2 (SMD: 0.24, 95% CI: 0.06–0.42) and combined intervention 3 (SMD: 0.43, 95% CI: 0.13–0.73) were significant in improving memory functioning in older adults compared to the control group. No statistically significant differences were observed in the outcomes for any of the other interventions.

**Figure 7.**
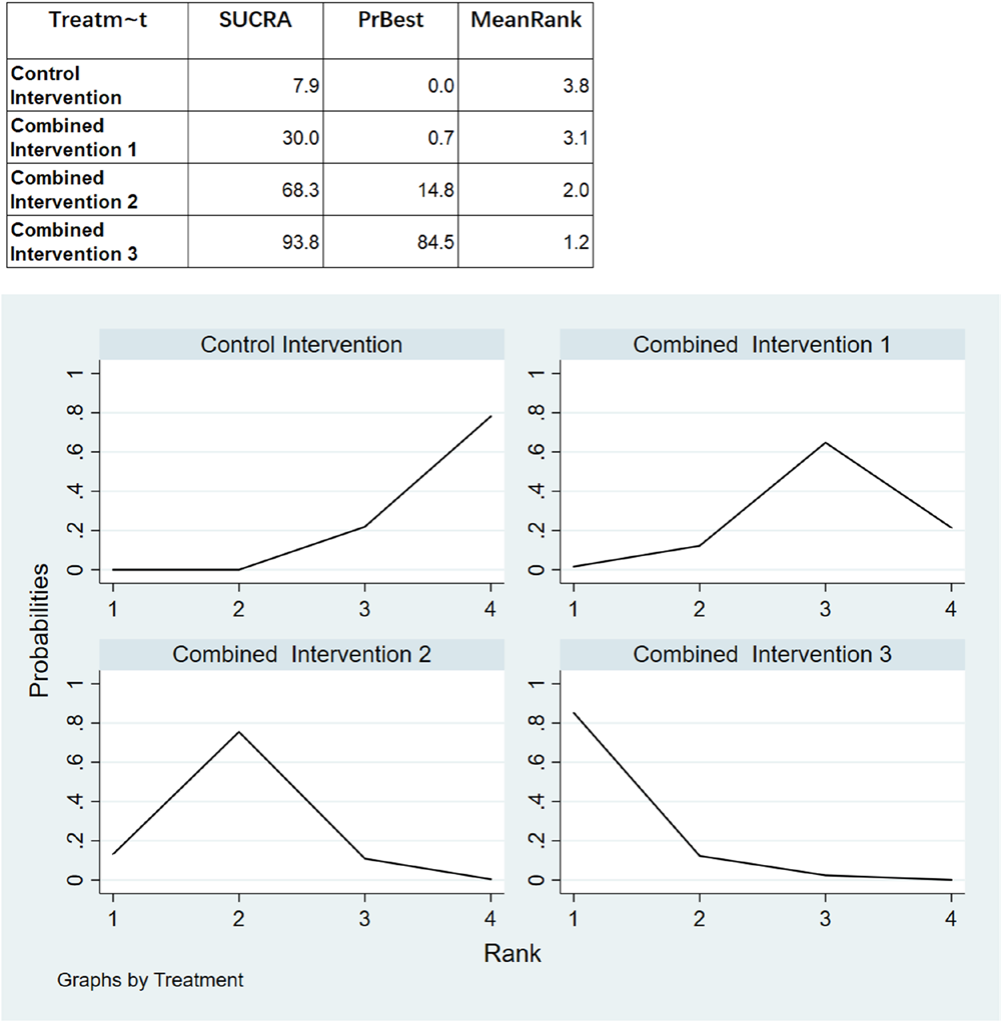
Results of the network ranking test for memory function.

**Figure 8.**
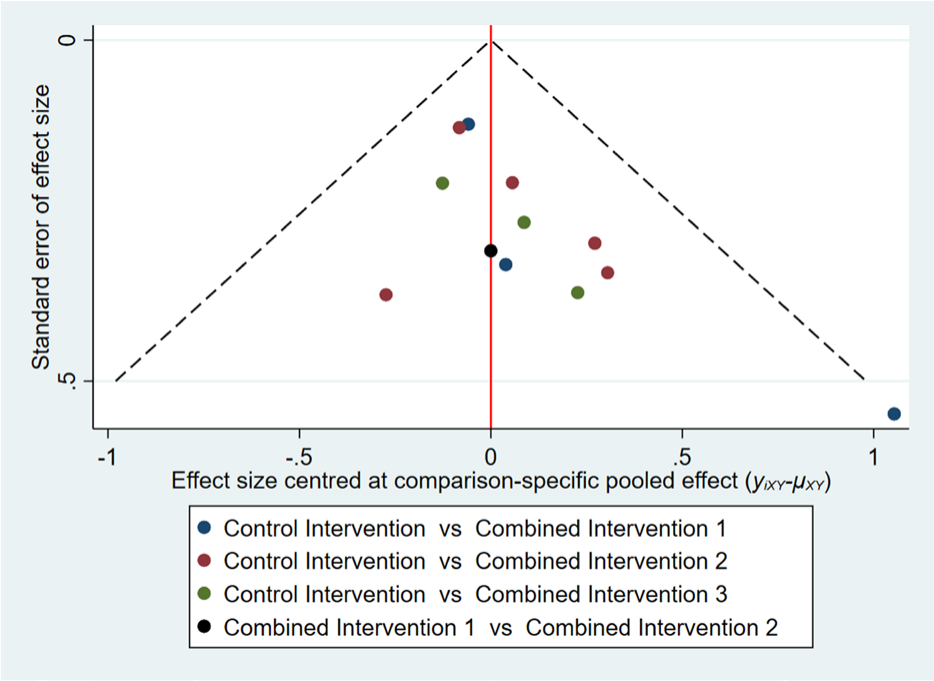
Comparison-adjusted funnel plot for memory function outcomes.

We ranked the superiority of all interventions using SUCRA. As shown in Figure 7, the probability that combined intervention 3 was the best intervention was 87.5%. Additionally, the SUCRA value of 93.8% for combined intervention 3 supports the assertion that this intervention mode is optimal. Publication bias of the studies included was detected using a comparison-adjusted funnel plot (Fig. 8). The comparison-adjusted funnel plot was largely symmetrical, indicating limited small sample effects and publication bias in the included studies.

**Figure 9.**
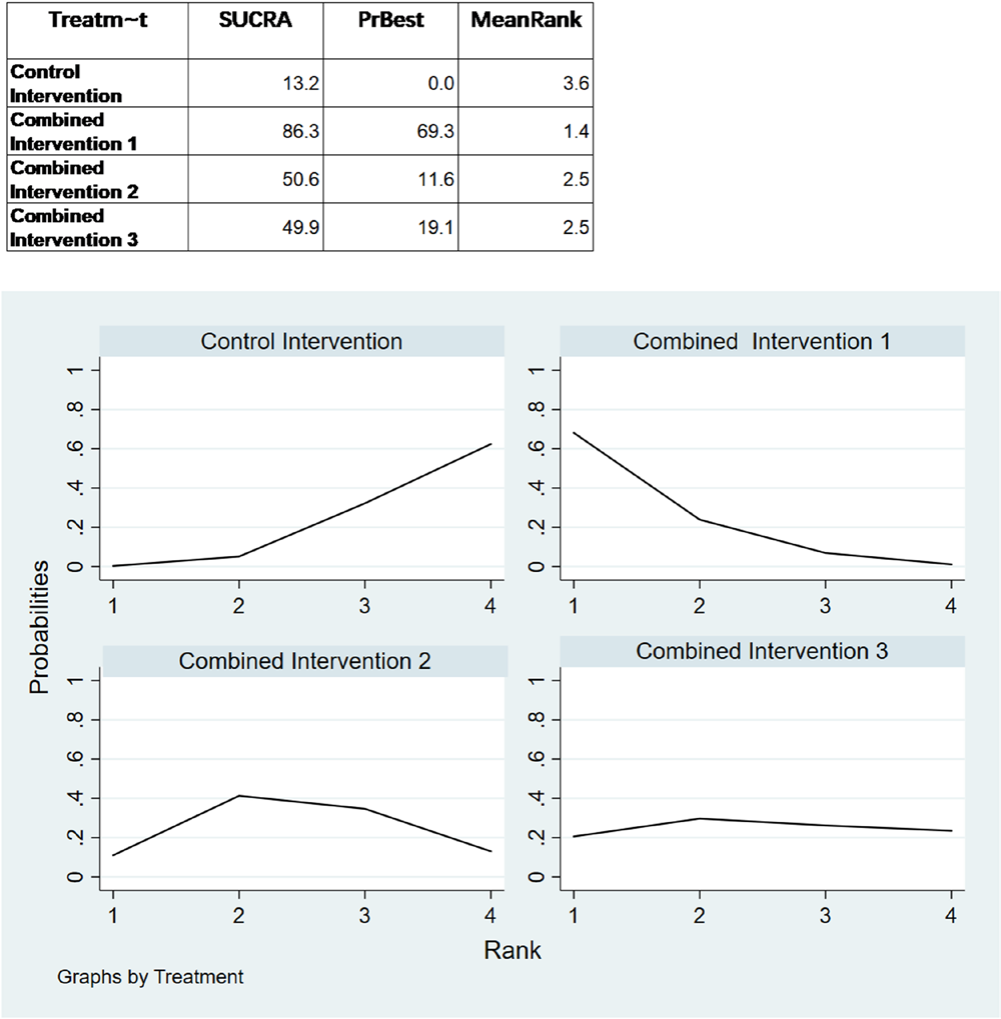
Results of the network ranking test for executive function.

**Figure 10.**
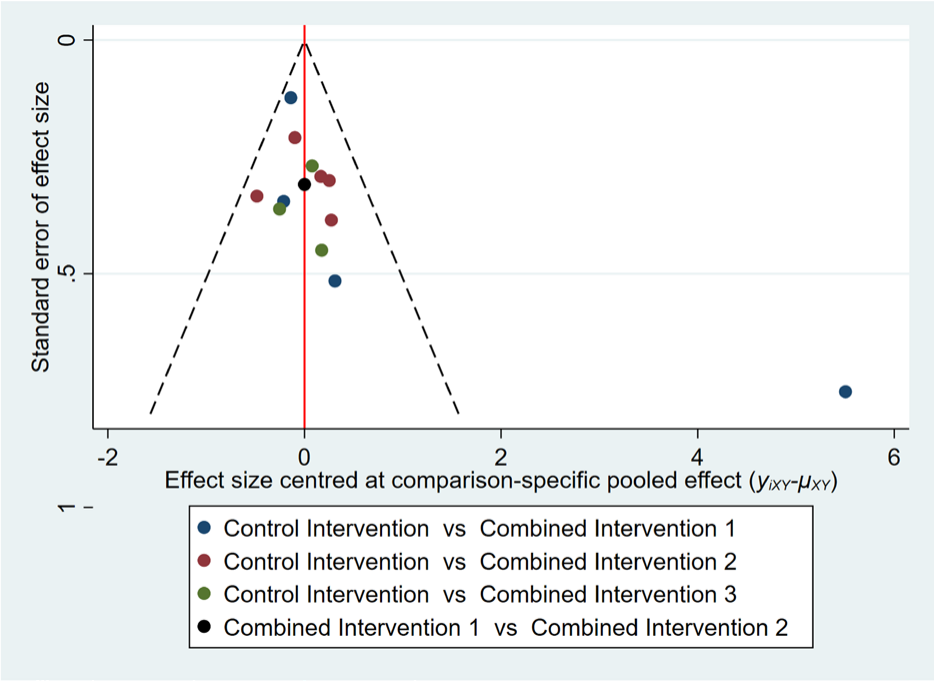
Comparison-adjusted funnel plot for executive function outcomes.

For the analysis of executive function through network meta-analysis, 13 out of 16 two-arm studies were included. Combined intervention 1, combined intervention 2, and combined intervention 3 were compared with the control group in 4, 5, and 3 studies, respectively. One study compared combined interventions 1 and 2. The results of the network meta-analysis showed (Fig. 6) that combined intervention 1 had a significant (SMD: 1.27, 95% CI: 0.05–2.49) improvement in executive function compared to the control group. The statistical significance of all other interventions was inconclusive. Nonetheless, Figure 9 illustrates that the combined intervention 1 had the highest probability of being the most effective treatment (69.3%) with a SUCRA value of 86.3%. When observing the comparison-adjusted funnel plots (Fig. 10), publication bias was detected in the included studies. Symmetry in the funnel plot was not achieved, with an outlier found outside the confidence interval. This indicates that there may be a small sample effect as well as publication bias in the studies.

### 3.7. Quality of evidence assessment

The CINeMA web application was used to evaluate the certainty of the results of the network meta-analyses. The comparison of combined intervention 2 and control, and combined intervention 3 and control in assessing memory function showed high levels of confidence. However, the confidence levels for the results comparing combined intervention 1 and 2, combine intervention 2 and 3, and outcomes were low. Moreover, the confidence levels in the results concerning all other memory and executive functions were low and extremely low, respectively (File S3, Supplemental Digital Content, http://links.lww.com/MD/O408).

## 4. Discussion

In older adults with cognitive health and mild cognitive dysfunction, including subjective cognitive decline and mild cognitive impairment, our network meta-analysis assessed the relative effectiveness of combined interventions including various exercise components for improving memory and executive function. To our knowledge, our study is the first to explore the difference among various cognitive-physical interventions or dual-task training based on the number of exercise components included to improve memory and executive function in older adults.

Our findings suggest that combined intervention 2 and combined intervention 3 significantly improved memory function in older adults. Based on the results of the network ranking, combined intervention 3 ranked highest with the greatest number of exercise components; combined intervention 2 ranked second; and combined intervention 1 ranked third. The network meta-analyses showed statistically significant differences in outcomes related with executive function only between combined intervention 1, which ranked highest, and the control group.

We believe that the results of memory function analyses may confirm the hypothesis that more the number of exercise components included in an intervention, the better would be the effect. Combined intervention 2 and combined intervention 3 had significant differences when compared to the control group, respectively, and the results of the network ranking test also supported that combined intervention 3 had the highest likelihood of being the first in terms of effectiveness. The results of pairwise and network meta-analyses were also generally consistent. The results of the quality of evidence assessment of memory function were favorable.

The results of the pairwise and the network meta-analyses varied considerably regarding the effects of the interventions on executive function, with very low confidence in the quality of their evidence, which makes us particularly cautious about interpreting the results. In the network meta-analysis, there was a significant difference observed between the combined intervention 1 and the control group, which may be attributed to the high heterogeneity and large sample size of the pairwise meta-analysis being compared. When pairwise meta-analysis and network meta-analysis results diverge, paired meta-analysis is generally considered to possess greater internal validity (and is indeed regarded as the highest level of evidence by certain guideline committees).^[34]^ Therefore, it is reasonable to believe that the pairwise meta-analyses with low heterogeneity showed that combined intervention 2 and combined intervention 3 were more effective than that in the control group.

In the meta-analysis of executive function, heterogeneity decreased significantly after omitting the study by Norouzi et al^[23]^ in the sensitivity analyses. In the comparison-adjusted funnel plot for executive function, the outlier at the lower right corner also pertains to this study.^[23]^ We analyzed the heterogeneity generated by combining the study by Norouzi et al with other studies and the reasons for publication bias. First, it is essential to note that participant blinding was not implemented in this study, which may have given rise to the influence of expectations and motivations on the results. Second, the small sample size in this study could lead to biased effect estimates, as the sample may not be representative of the broader population, and random errors could be more pronounced.

As this study is the first to investigate the impact of varying numbers of exercise components in a combined cognitive-physical intervention program to improve the cognitive function in older adults, no prior meta-analysis aligns with our findings. However, a number of studies have examined the effects of interventions combining exercise and cognitive interventions to improve cognitive function in older adults. A systematic review by Li et al^[35]^ assessed the effects of Tai Chi and cognitive interventions on older adults with or without cognitive impairment, finding significant improvements in memory. However, it remains unclear whether the combined intervention is superior to single interventions. Notably, this systematic review included only 6 studies on cognitive outcomes, which may limit statistical power and introduce potential selection bias. A meta-analysis by Guo et al^[36]^ included 21 studies investigating the effects of cognitive-physical interventions on executive function in older adults. While the results showed that cognitive-physical combined interventions yielded significant effects compared to control groups, they were not superior to single interventions. On one hand, some of the studies included had short intervention durations, which may not have been sufficient to produce significant effects. On the other hand, high-intensity interventions may lead to excessive stress and cognitive fatigue in older adults, which could negatively influence the effect size.^[37]^ A recent meta-analysis conducted by Meng et al^[38]^ integrated 16 studies investigating the impact of combined physical exercise and cognitive interventions on cognitive function in older adults with MCI. Their analysis found that the combined intervention significantly affected both memory and executive function, and was more effective than either physical exercise or cognitive training alone. While these findings are intriguing, the study population was limited to older adults with MCI. Therefore, it can be inferred that the combined intervention indeed offers benefits compared to the control group. However, further research is needed to ascertain the effects between combined intervention and single intervention, as well as among different combinations of interventions.

Previous overall evidence suggests that physical exercise may influence gray matter structures, including the hippocampus.^[39]^ Emerging evidence further indicates that exercise exerts additional neuroplastic effects, such as enhancing white matter integrity and myelination, as well as improving neurovascular function, neuronal connectivity, and activation.^[39]^ Cognitive stimulation and training have been shown to enhance cognitive reserve and promote neuroplastic changes. This implies that targeted cognitive activities can activate neural networks in the brain, leading to the formation of new connections or the strengthening of existing ones, thereby enhancing cognitive function.^[40]^ Currently, there is ambiguity concerning the mechanisms of combined cognitive and physical interventions. The article by Tao et al^[41]^ outlines 2 possible mechanisms: the reciprocal stimulation of neurogenic plasticity. In addition, cognitive-physical interventions may provide complementary benefits in increased neurogenesis, synapse formation, promotion of cerebral vascular regeneration, increased blood flow, and enhanced plasticity in the aging brain.^[42,43]^ Physical training is believed to improve synaptic plasticity and cell proliferation, while cognitive training directs the creation of synapses between these newly-formed neurons and preexisting neural networks.^[44,45]^ Interventions that incorporate multiple exercise components may enhance the effectiveness of exercise training by establishing interconnectivity across molecular, cellular, organ, and systemic levels, thereby elevating the efficacy of cognitive-physical interventions.

At present, non-pharmacological interventions for cognitive decline in older adults predominantly include lifestyle modifications, such as physical activity, healthy diet, and social engagement, alongside brain exercises like cognitive training. Our study underscores the importance of incorporating a diverse range of physical activities in cognitive-physical combined interventions. We recommend structuring physical activity interventions in a multi-component approach. It is also essential to tailor interventions according to the individual health status of older adults. Clinicians and healthcare professionals should consider factors such as physical condition and cognitive status when selecting and designing interventions, in order to maximize improvements in cognitive health.

## 5. Limitations

First, owing to the limited availability of studies for each intervention, it was difficult to analyze other indicators of cognitive functions (e.g., global cognitive functions) and determine potential confounders (e.g., frequency duration, and intensity of the intervention). Therefore, our results should be interpreted with caution. Second, the heterogeneity in the cognitive states of the participants across the included studies may have affected the interpretation of the results. This review included older adults with cognitive health, subjective cognitive decline, and mild cognitive impairment. Therefore, the distribution of benefits of combined interventions may not have been equally beneficial for older adults with different cognitive statuses. Third, while this study did not specifically address health inequities, future research could investigate how health disparities may influence the effectiveness of interventions across different populations. Finally, the difference between the intervention modalities included in the study was the number of other exercise components in the combined intervention; we did not consider the type of exercise component. Future studies could further explore the potential impact of the kind of exercise component.

## 6. Conclusion

In the future, cognitive-physical interventions for older adults may benefit from the development and testing of multi-component exercise programs. Future research should focus on identifying the optimal combination of one or more types of physical exercises with cognitive tasks, as well as exploring how to tailor interventions to individual needs in order to optimize cognitive health outcomes.

## Acknowledgments

We thank Bullet Edits Limited for the linguistic editing and proofreading of the manuscript.

## Author contributions

**Conceptualization:** Jiawei Liu.

**Data curation:** Conglin Han.

**Methodology:** Dan Zhang, Jiawei Liu.

**Software:** Ruifeng Sun.

**Supervision:** Weishuang Sun.

**Validation:** Weishuang Sun.

**Writing – original draft:** Conglin Han.

**Writing – review & editing:** WeiJun Gong.

## Supplementary Material


