# Supplementary material for: Are more exercise components in combined cognitive and physical training better for older adults?: A systematic review and network meta-analysis of randomized controlled trials
Source: Medicine (Baltimore). 2025 Feb 21;104(8):e41572. doi: 10.1097/MD.0000000000041572 (PMC11857035; doi:10.1097/MD.0000000000041572)
Supplement: Supplementary file 1 [file medi-104-e41572-s001.docx]

**Pubmed Search strategy**

| Search number | Search Details | Results |
| --- | --- | --- |
| 1# | "Aged"[MeSH Terms] OR "Elderly"[Title/Abstract] OR "older adult"[Title/Abstract] OR "senior"[Title/Abstract] | 3578025 |
| 2# | "Cognitive Dysfunction"[MeSH Terms] OR "cognitive dysfunctions"[Title/Abstract] OR "dysfunction cognitive"[Title/Abstract] OR "cognitive impairment*"[Title/Abstract] OR "impairment cognitive"[Title/Abstract] OR "cognitive disorder*"[Title/Abstract] OR "disorder cognitive"[Title/Abstract] OR "mild cognitive impairment*"[Title/Abstract] OR "cognitive impairment mild"[Title/Abstract] OR "impairment mild cognitive"[Title/Abstract] OR "cognitive decline*"[Title/Abstract] OR "decline cognitive"[Title/Abstract] OR "mental deterioration*"[Title/Abstract] OR "deterioration mental"[Title/Abstract] | 130401 |
| 3# | "Aged"[MeSH Terms] OR "Elderly"[Title/Abstract] OR "older adult"[Title/Abstract] OR "senior"[Title/Abstract] OR "Cognitive Dysfunction"[MeSH Terms] OR "cognitive dysfunctions"[Title/Abstract] OR "dysfunction cognitive"[Title/Abstract] OR "cognitive impairment*"[Title/Abstract] OR "impairment cognitive"[Title/Abstract] OR "cognitive disorder*"[Title/Abstract] OR "disorder cognitive"[Title/Abstract] OR "mild cognitive impairment*"[Title/Abstract] OR "cognitive impairment mild"[Title/Abstract] OR "impairment mild cognitive"[Title/Abstract] OR "cognitive decline*"[Title/Abstract] OR "decline cognitive"[Title/Abstract] OR "mental deterioration*"[Title/Abstract] OR "deterioration mental"[Title/Abstract] | 3655827 |
| 4# | "combined"[Title/Abstract] OR "combination"[Title/Abstract] OR "multimodal"[Title/Abstract] OR "dual-task"[Title/Abstract] OR "dual-task"[Title/Abstract] OR "multidomain"[Title/Abstract] OR "multicomponent"[Title/Abstract] OR "multi-modal"[Title/Abstract] OR "multi-domain"[Title/Abstract] OR "multi-component"[Title/Abstract] | 1951431 |
| 5# | "Exercise"[MeSH Terms] OR "exercise*"[Title/Abstract] OR "physical activity"[Title/Abstract] OR "activities physical"[Title/Abstract] OR "activity physical"[Title/Abstract] OR "physical activities"[Title/Abstract] OR "exercise physical"[Title/Abstract] OR "physical exercise*"[Title/Abstract] OR "acute exercise*"[Title/Abstract] OR "exercise acute"[Title/Abstract] OR "exercise isometric"[Title/Abstract] OR "isometric exercise*"[Title/Abstract] OR "exercise aerobic"[Title/Abstract] OR "aerobic exercise*"[Title/Abstract] OR "exercise training*"[Title/Abstract] OR "training exercise"[Title/Abstract] | 568312 |
| 6# | (("cognition"[MeSH Terms] OR "cognition"[All Fields] OR "cognitions"[All Fields] OR "cognitive"[All Fields] OR "cognitively"[All Fields] OR "cognitives"[All Fields]) AND "training*"[All Fields]) OR (("cognition"[MeSH Terms] OR "cognition"[All Fields] OR "cognitions"[All Fields] OR "cognitive"[All Fields] OR "cognitively"[All Fields] OR "cognitives"[All Fields]) AND "training*"[All Fields]) OR ("training"[All Fields] AND ("brain"[MeSH Terms] OR "brain"[All Fields] OR "brains"[All Fields] OR "brain s"[All Fields])) OR ("training"[All Fields] AND ("cognition"[MeSH Terms] OR "cognition"[All Fields] OR "cognitions"[All Fields] OR "cognitive"[All Fields] OR "cognitively"[All Fields] OR "cognitives"[All Fields])) OR ("cognitive training"[MeSH Terms] OR ("cognitive"[All Fields] AND "training"[All Fields]) OR "cognitive training"[All Fields] OR ("cognitive"[All Fields] AND "rehabilitation"[All Fields]) OR "cognitive rehabilitation"[All Fields]) OR ("cognitive training"[MeSH Terms] OR ("cognitive"[All Fields] AND "training"[All Fields]) OR "cognitive training"[All Fields] OR ("rehabilitation"[All Fields] AND "cognitive"[All Fields]) OR "rehabilitation cognitive"[All Fields]) OR (("memories"[All Fields] OR "memory"[MeSH Terms] OR "memory"[All Fields] OR "memory s"[All Fields]) AND "training*"[All Fields]) OR ("training"[All Fields] AND ("memories"[All Fields] OR "memory"[MeSH Terms] OR "memory"[All Fields] OR "memory s"[All Fields])) OR (("cognition"[MeSH Terms] OR "cognition"[All Fields] OR "cognitions"[All Fields] OR "cognitive"[All Fields] OR "cognitively"[All Fields] OR "cognitives"[All Fields]) AND "exercise*"[All Fields]) OR (("brain"[MeSH Terms] OR "brain"[All Fields] OR "brains"[All Fields] OR "brain s"[All Fields]) AND "exercise*"[All Fields]) OR (("memories"[All Fields] OR "memory"[MeSH Terms] OR "memory"[All Fields] OR "memory s"[All Fields]) AND "exercise*"[All Fields]) OR (("reasonableness"[All Fields] OR "reasoned"[All Fields] OR "reasoner"[All Fields] OR "reasoners"[All Fields] OR "reasoning"[All Fields] OR "reasonings"[All Fields]) AND "exercise*"[All Fields]) OR (("cognition"[MeSH Terms] OR "cognition"[All Fields] OR "cognitions"[All Fields] OR "cognitive"[All Fields] OR "cognitively"[All Fields] OR "cognitives"[All Fields]) AND "stimulation*"[All Fields]) OR (("memories"[All Fields] OR "memory"[MeSH Terms] OR "memory"[All Fields] OR "memory s"[All Fields]) AND "stimulation*"[All Fields]) OR (("memories"[All Fields] OR "memory"[MeSH Terms] OR "memory"[All Fields] OR "memory s"[All Fields]) AND ("enhance"[All Fields] OR "enhanced"[All Fields] OR "enhancement"[All Fields] OR "enhancements"[All Fields] OR "enhancer"[All Fields] OR "enhancer s"[All Fields] OR "enhancers"[All Fields] OR "enhances"[All Fields] OR "enhancing"[All Fields])) OR (("cognition"[MeSH Terms] OR "cognition"[All Fields] OR "cognitions"[All Fields] OR "cognitive"[All Fields] OR "cognitively"[All Fields] OR "cognitives"[All Fields]) AND ("enhance"[All Fields] OR "enhanced"[All Fields] OR "enhancement"[All Fields] OR "enhancements"[All Fields] OR "enhancer"[All Fields] OR "enhancer s"[All Fields] OR "enhancers"[All Fields] OR "enhances"[All Fields] OR "enhancing"[All Fields])) | 274413 |
| 7# | ("Aged"[MeSH Terms] OR "Elderly"[Title/Abstract] OR "older adult"[Title/Abstract] OR "senior"[Title/Abstract] OR ("Cognitive Dysfunction"[MeSH Terms] OR ("cognitive dysfunctions"[Title/Abstract] OR "dysfunction cognitive"[Title/Abstract] OR "cognitive impairment*"[Title/Abstract] OR "impairment cognitive"[Title/Abstract] OR "cognitive disorder*"[Title/Abstract] OR "disorder cognitive"[Title/Abstract] OR "mild cognitive impairment*"[Title/Abstract] OR "cognitive impairment mild"[Title/Abstract] OR "impairment mild cognitive"[Title/Abstract] OR "cognitive decline*"[Title/Abstract] OR "decline cognitive"[Title/Abstract] OR "mental deterioration*"[Title/Abstract] OR "deterioration mental"[Title/Abstract]))) AND ((("cognition"[MeSH Terms] OR "cognition"[All Fields] OR "cognitions"[All Fields] OR "Cognitive"[All Fields] OR "cognitively"[All Fields] OR "cognitives"[All Fields]) AND "training*"[All Fields]) OR (("cognition"[MeSH Terms] OR "cognition"[All Fields] OR "cognitions"[All Fields] OR "Cognitive"[All Fields] OR "cognitively"[All Fields] OR "cognitives"[All Fields]) AND "training*"[All Fields]) OR ("training"[All Fields] AND ("brain"[MeSH Terms] OR "brain"[All Fields] OR "brains"[All Fields] OR "brain s"[All Fields])) OR ("training"[All Fields] AND ("cognition"[MeSH Terms] OR "cognition"[All Fields] OR "cognitions"[All Fields] OR "Cognitive"[All Fields] OR "cognitively"[All Fields] OR "cognitives"[All Fields])) OR ("cognitive training"[MeSH Terms] OR ("Cognitive"[All Fields] AND "training"[All Fields]) OR "cognitive training"[All Fields] OR ("Cognitive"[All Fields] AND "rehabilitation"[All Fields]) OR "cognitive rehabilitation"[All Fields]) OR ("cognitive training"[MeSH Terms] OR ("Cognitive"[All Fields] AND "training"[All Fields]) OR "cognitive training"[All Fields] OR ("rehabilitation"[All Fields] AND "Cognitive"[All Fields]) OR "rehabilitation cognitive"[All Fields]) OR (("memories"[All Fields] OR "memory"[MeSH Terms] OR "memory"[All Fields] OR "memory s"[All Fields]) AND "training*"[All Fields]) OR ("training"[All Fields] AND ("memories"[All Fields] OR "memory"[MeSH Terms] OR "memory"[All Fields] OR "memory s"[All Fields])) OR (("cognition"[MeSH Terms] OR "cognition"[All Fields] OR "cognitions"[All Fields] OR "Cognitive"[All Fields] OR "cognitively"[All Fields] OR "cognitives"[All Fields]) AND "exercise*"[All Fields]) OR (("brain"[MeSH Terms] OR "brain"[All Fields] OR "brains"[All Fields] OR "brain s"[All Fields]) AND "exercise*"[All Fields]) OR (("memories"[All Fields] OR "memory"[MeSH Terms] OR "memory"[All Fields] OR "memory s"[All Fields]) AND "exercise*"[All Fields]) OR (("reasonableness"[All Fields] OR "reasoned"[All Fields] OR "reasoner"[All Fields] OR "reasoners"[All Fields] OR "reasoning"[All Fields] OR "reasonings"[All Fields]) AND "exercise*"[All Fields]) OR (("cognition"[MeSH Terms] OR "cognition"[All Fields] OR "cognitions"[All Fields] OR "Cognitive"[All Fields] OR "cognitively"[All Fields] OR "cognitives"[All Fields]) AND "stimulation*"[All Fields]) OR (("memories"[All Fields] OR "memory"[MeSH Terms] OR "memory"[All Fields] OR "memory s"[All Fields]) AND "stimulation*"[All Fields]) OR (("memories"[All Fields] OR "memory"[MeSH Terms] OR "memory"[All Fields] OR "memory s"[All Fields]) AND ("enhance"[All Fields] OR "enhanced"[All Fields] OR "enhancement"[All Fields] OR "enhancements"[All Fields] OR "enhancer"[All Fields] OR "enhancer s"[All Fields] OR "enhancers"[All Fields] OR "enhances"[All Fields] OR "enhancing"[All Fields])) OR (("cognition"[MeSH Terms] OR "cognition"[All Fields] OR "cognitions"[All Fields] OR "Cognitive"[All Fields] OR "cognitively"[All Fields] OR "cognitives"[All Fields]) AND ("enhance"[All Fields] OR "enhanced"[All Fields] OR "enhancement"[All Fields] OR "enhancements"[All Fields] OR "enhancer"[All Fields] OR "enhancer s"[All Fields] OR "enhancers"[All Fields] OR "enhances"[All Fields] OR "enhancing"[All Fields]))) AND ("Exercise"[MeSH Terms] OR ("exercise*"[Title/Abstract] OR "physical activity"[Title/Abstract] OR "activities physical"[Title/Abstract] OR "activity physical"[Title/Abstract] OR "physical activities"[Title/Abstract] OR "exercise physical"[Title/Abstract] OR "physical exercise*"[Title/Abstract] OR "acute exercise*"[Title/Abstract] OR "exercise acute"[Title/Abstract] OR "exercise isometric"[Title/Abstract] OR "isometric exercise*"[Title/Abstract] OR "exercise aerobic"[Title/Abstract] OR "aerobic exercise*"[Title/Abstract] OR "exercise training*"[Title/Abstract] OR "training exercise"[Title/Abstract])) AND ("combined"[Title/Abstract] OR "combination"[Title/Abstract] OR "multimodal"[Title/Abstract] OR "dual-task"[Title/Abstract] OR "dual-task"[Title/Abstract] OR "multidomain"[Title/Abstract] OR "multicomponent"[Title/Abstract] OR "multi-modal"[Title/Abstract] OR "multi-domain"[Title/Abstract] OR "multi-component"[Title/Abstract]) | 2077 |

**Embase Search strategy**

| Search number | Search Details | Results |
| --- | --- | --- |
| 1# | 'aged'/exp OR aged OR 'older adults'/exp OR 'older adults' | 5783146 |
| 2# | 'cognitive defect'/exp OR 'cognitive defect' OR 'cognitive decline'/exp OR 'cognitive decline' OR (cognitive AND ('decline'/exp OR decline)) OR 'mental deterioration'/exp OR 'mental deterioration' | 640484 |
| 3# | 'exercise'/exp OR exercise OR 'physical activity'/exp OR 'physical activity' OR 'aerobic exercise'/exp OR 'aerobic exercise' OR 'acute exercise'/exp OR 'acute exercise' OR 'isometric exercise'/exp OR 'isometric exercise' OR (isometric AND ('exercise'/exp OR exercise)) | 1152438 |
| 4# | combined OR combination OR multimodal OR (dual AND ('task'/exp OR task)) OR 'dual task'/exp OR 'dual task' OR multidomain OR 'multi domain' OR 'multi component' OR multicomponent | 3438346 |
| 5# | 'cognitive training'/exp OR 'cognitive training' OR (cognitive AND ('training'/exp OR training)) OR 'brain training'/exp OR 'brain training' OR (('brain'/exp OR brain) AND ('training'/exp OR training)) OR 'cognitive rehabilitation'/exp OR 'cognitive rehabilitation' OR (cognitive AND ('rehabilitation'/exp OR rehabilitation)) OR 'brain rehabilitation' OR (('brain'/exp OR brain) AND ('rehabilitation'/exp OR rehabilitation)) OR 'memory training'/exp OR 'memory training' OR (('memory'/exp OR memory) AND ('training'/exp OR training)) OR 'cognitive exercise'/exp OR 'cognitive exercise' OR (cognitive AND ('exercise'/exp OR exercise)) OR 'brain exercise' OR (('brain'/exp OR brain) AND ('exercise'/exp OR exercise)) OR 'memory exercise' OR (('memory'/exp OR memory) AND ('exercise'/exp OR exercise)) OR 'cognitive stimulation'/exp OR 'cognitive stimulation' OR (cognitive AND ('stimulation'/exp OR stimulation)) OR 'memory stimulation' OR (('memory'/exp OR memory) AND ('stimulation'/exp OR stimulation)) OR 'memory enhancement'/exp OR 'memory enhancement' OR (('memory'/exp OR memory) AND ('enhancement'/exp OR enhancement)) OR 'cognitive enhancement'/exp OR 'cognitive enhancement' OR (cognitive AND ('enhancement'/exp OR enhancement)) | 427219 |
| 6# | #1 OR #2 | 6209554 |
| 7# | #3 AND #4 AND #5 AND #6 | 5479 |

**Cochrane Library Search strategy**

| Search number | Search Details | Results |
| --- | --- | --- |
| 1# | MeSH descriptor: [Cognition Disorders] explode all trees | 7279 |
| 2# | 'cognitive defect'/exp OR 'cognitive defect' OR 'cognitive decline'/exp OR 'cognitive decline' OR (cognitive AND ('decline'/exp OR decline)) OR 'mental deterioration'/exp OR 'mental deterioration' | 1192 |
| 3# | #1 OR #2 | 8379 |
| 4# | MeSH descriptor: [Exercise] explode all trees | 38509 |
| 5# | (Exercise*, Isometric):ti,ab,kw OR (Physical Exercise*):ti,ab,kw OR (Physical Activit*):ti,ab,kw OR (Aerobic Exercise*):ti,ab,kw AND (Training*, Exercise):ti,ab,kw (Word variations have been searched) | 87419 |
| 6# | #4 OR #5 | 107512 |
| 7# | (dual-task):ti,ab,kw OR (combination):ti,ab,kw OR (combined):ti,ab,kw OR (multimodal):ti,ab,kw OR (multicomponent):ti,ab,kw (Word variations have been searched) | 324632 |
| 8# | MeSH descriptor: [Cognitive Training] explode all trees | 48 |
| 9# | (Cognitive Rehabilitation OR Rehabilitation, Cognitive):ti,ab,kw OR (Training, Brain OR Brain Training):ti,ab,kw OR (Training, Cognitive):ti,ab,kw OR (Memory Training OR Training, Memory):ti,ab,kw (Word variations have been searched) | 32260 |
| 10# | #8 OR #9 | 32260 |
| 11# | #6 AND #7 AND #10 | 2145 |
| 12# | MeSH descriptor: [Aged] explode all trees | 255705 |
| 13# | #3 OR #12 | 260699 |
| 14# | #11 AND #13 | 620 |
