# Supplementary material for: Are more exercise components in combined cognitive and physical training better for older adults?: A systematic review and network meta-analysis of randomized controlled trials
Source: Medicine (Baltimore). 2025 Feb 21;104(8):e41572. doi: 10.1097/MD.0000000000041572 (PMC11857035; doi:10.1097/MD.0000000000041572)

**Sensitivity analysis**

| CP 1 VS Control group（Executive function） | | |  |  | |  |  |  |
| --- | --- | --- | --- | --- | --- | --- | --- | --- |
|  | | | Heterogeneity | | | | | |
| Study omitted | Standardized mean difference | p-value | Tau² | Chi² | df (degrees of freedom) | | P-value | I²(%) |
| C. Fabre 2002 | 1.84[-0.17,3.85] | 0.07 | 2.94 | 54.88 | | 2 | < 0.00001 | 96% |
| Combourieu Donnezan, Laure 2018 | 2.10[-0.52,4.73] | 0.12 | 5..1 | 54.84 | | 2 | < 0.00001 | 96% |
| Lam, L C 2015 | 2.11[-0.75,4.96] | 0.10 | 6.06 | 48.09 | | 2 | < 0.00001 | 96% |
| Norouzi, E 2019 | 0.10[-0.04,0.41] | 0.12 | 0 | 0.79 | | 2 | 0.67 | 0% |

**Inconsistency analysis of network mate-analysis(memory function)**


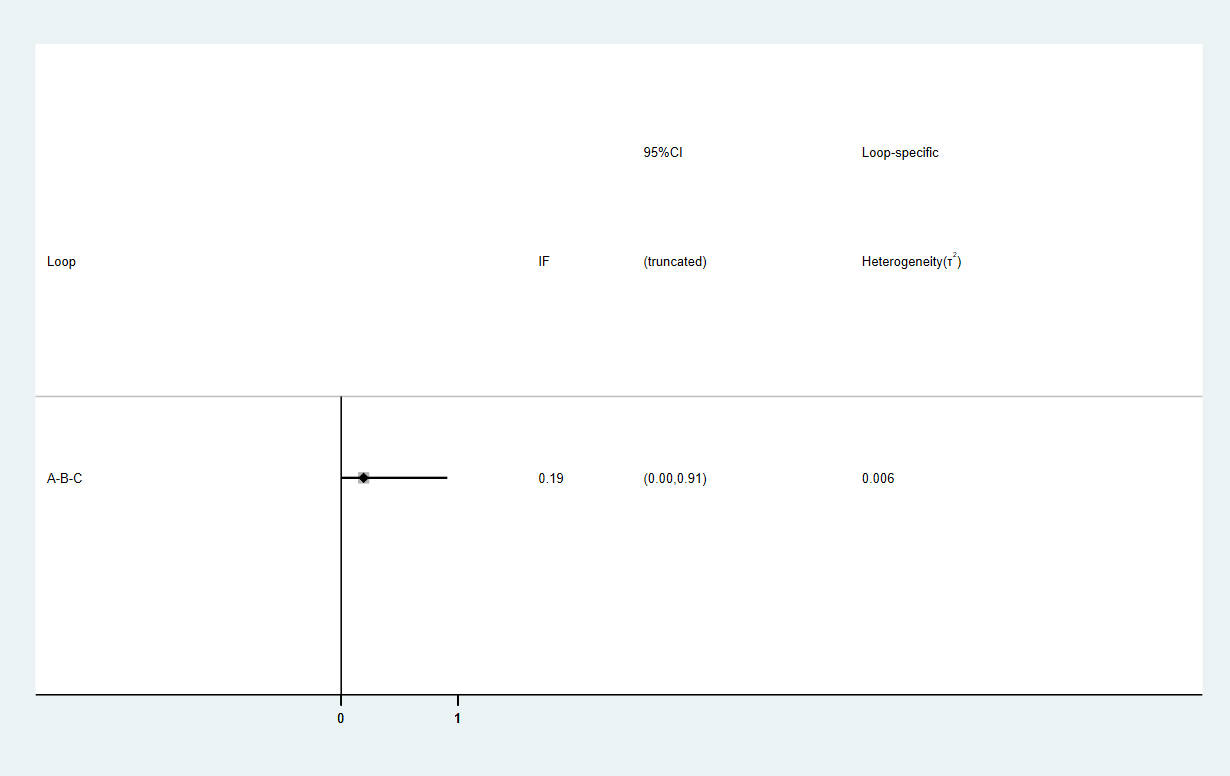


**Inconsistency analysis of network mate-analysis(executive function)**


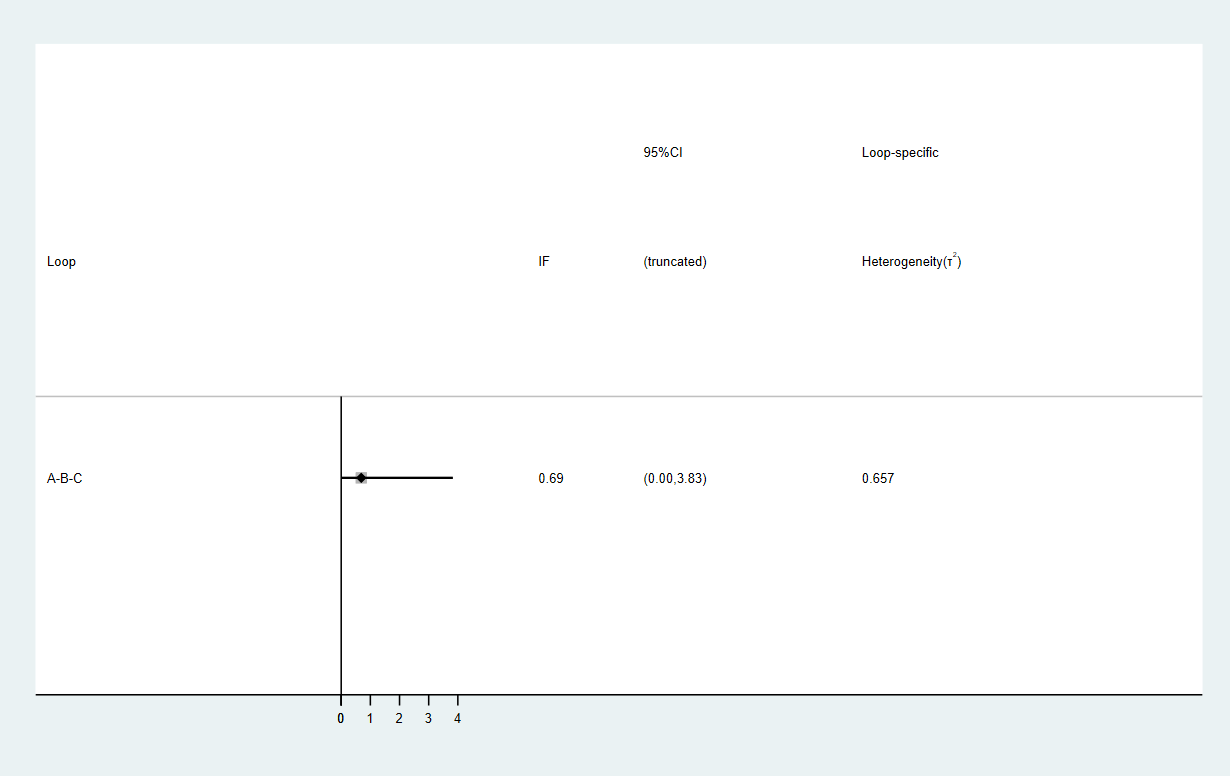

Supplement: Supplementary file 2 [file medi-104-e41572-s002.docx]
