# Supplementary material for: Are more exercise components in combined cognitive and physical training better for older adults?: A systematic review and network meta-analysis of randomized controlled trials
Source: Medicine (Baltimore). 2025 Feb 21;104(8):e41572. doi: 10.1097/MD.0000000000041572 (PMC11857035; doi:10.1097/MD.0000000000041572)
Supplement: Supplementary file 3 [file medi-104-e41572-s003.pdf]

Memory Function(A: control intervention, B: combined intervention 1, C: combined intervention 2, D: combined intervention 3)

Comparison	Number of studies	Within-study bias	Reporting bias	Indirectness	Imprecision	Heterogeneity	Incoherence	Confidence rating	Reason(s) for downgrading
A:B	3	Some concerns	Low risk	No concerns	Major concerns	No concerns	No concerns	Very low	["Within-study bias","Imprecision"]
A:C	5	No concerns	Low risk	No concerns	No concerns	No concerns	No concerns	High	[]
A:D	3	No concerns	Low risk	No concerns	No concerns	No concerns	No concerns	High	[]
B:C	1	No concerns	Low risk	No concerns	Major concerns	No concerns	No concerns	Low	["Imprecision"]
B:D	0	Some concerns	Low risk	No concerns	Major concerns	No concerns	No concerns	Very low	["Within-study bias","Imprecision"]
C:D	0	No concerns	Low risk	No concerns	Major concerns	No concerns	No concerns	Low	["Imprecision"]

Executive Function(A: control intervention, B: combined intervention 1, C: combined intervention 2, D: combined intervention 3)

Comparison	Number of studies	Within-study bias	Reporting bias	Indirectness	Imprecision	Heterogeneity	Incoherence	Confidence rating	Reason(s) for downgrading
A:B	4	Some concerns	Some concerns	No concerns	No concerns	Major concerns	No concerns	Very low	["Within-study bias","Reporting bias","Heterogeneity"]
A:C	5	Some concerns	Some concerns	No concerns	Major concerns	No concerns	No concerns	Very low	["Within-study bias","Reporting bias","Imprecision"]
A:D	3	Some concerns	Some concerns	No concerns	Major concerns	No concerns	No concerns	Very low	["Within-study bias","Reporting bias","Imprecision"]
B:C	1	Some concerns	Some concerns	No concerns	Major concerns	No concerns	No concerns	Very low	["Within-study bias","Reporting bias","Imprecision"]
B:D	0	Some concerns	Some concerns	No concerns	Major concerns	No concerns	No concerns	Very low	["Within-study bias","Reporting bias","Imprecision"]
C:D	0	Some concerns	Some concerns	No concerns	Major concerns	No concerns	No concerns	Very low	["Within-study bias","Reporting bias","Imprecision"]
